# Microarray analyses reveal strain-specific antibody responses to *Plasmodium falciparum* apical membrane antigen 1 variants following natural infection and vaccination

**DOI:** 10.1038/s41598-020-60551-z

**Published:** 2020-03-03

**Authors:** Jason A. Bailey, Andrea A. Berry, Mark A. Travassos, Amed Ouattara, Sarah Boudova, Emmanuel Y. Dotsey, Andrew Pike, Christopher G. Jacob, Matthew Adams, John C. Tan, Ryan M. Bannen, Jigar J. Patel, Jozelyn Pablo, Rie Nakajima, Algis Jasinskas, Sheetij Dutta, Shannon Takala-Harrison, Kirsten E. Lyke, Matthew B. Laurens, Amadou Niangaly, Drissa Coulibaly, Bourema Kouriba, Ogobara K. Doumbo, Mahamadou A. Thera, Philip L. Felgner, Christopher V. Plowe

**Affiliations:** 1 Center for Vaccine Development and Global Health, University of Maryland School of Medicine, Baltimore, MD USA; 2 Department of Physiology & Biophysics, University of California, Irvine, CA USA; 3 Wellcome Sanger Institute, Hinxton, United Kingdom; 4 Previous address: Roche Sequencing Solutions, Madison, WI USA; 5 Present Address: Nimble Therapeutics, Madison, WI USA; 6 U.S. Military Malaria Vaccine Program, Walter Reed Army Institute of Research, Silver Spring, MD USA; 7 Malaria Research and Training Center, University of Sciences, Techniques and Technologies of Bamako, Bamako, Mali; 8 Duke Global Health Institute, Duke University, Durham, NC USA

**Keywords:** Antibodies, Protein vaccines

## Abstract

Vaccines based on *Plasmodium falciparum* apical membrane antigen 1 (AMA1) have failed due to extensive polymorphism in AMA1. To assess the strain-specificity of antibody responses to malaria infection and AMA1 vaccination, we designed protein and peptide microarrays representing hundreds of unique AMA1 variants. Following clinical malaria episodes, children had short-lived, sequence-independent increases in average whole-protein seroreactivity, as well as strain-specific responses to peptides representing diverse epitopes. Vaccination resulted in dramatically increased seroreactivity to all 263 AMA1 whole-protein variants. High-density peptide analysis revealed that vaccinated children had increases in seroreactivity to four distinct epitopes that exceeded responses to natural infection. A single amino acid change was critical to seroreactivity to peptides in a region of AMA1 associated with strain-specific vaccine efficacy. Antibody measurements using whole antigens may be biased towards conserved, immunodominant epitopes. Peptide microarrays may help to identify immunogenic epitopes, define correlates of vaccine protection, and measure strain-specific vaccine-induced antibodies.

## Introduction

*Plasmodium falciparum* apical membrane antigen 1 (AMA1) is a malaria parasite surface protein involved in red blood cell invasion^1^. AMA1 has been a leading target for vaccine development because of its high antigenicity and the ability of AMA1 antibodies to inhibit parasite growth both *in vitro* and in non-human primates^2–6^. However, *in vitro* and molecular epidemiological studies provided early evidence that AMA1 exhibits immune evasion that is both domain- and sequence-specific^7–12^. The AMA1 protein contains a hydrophobic cleft that is the binding site of red blood cell invasion machinery^13–16^. Synthetic peptides that bind to this hydrophobic cleft prevent interaction with the red blood cell invasion complex, obstructing parasite invasion^17,18^. The hydrophobic cleft is in the first of AMA1’s three domains, surrounded by six flexible, polymorphic loops thought to prevent host antibodies from disrupting the formation of the invasion complex^16,19^. Moreover, polymorphisms in a region known as the cluster 1 loop of domain 1 have been shown to affect the binding of inhibitory monoclonal antibodies *in vitro*^7,20^. A monovalent AMA1 subunit vaccine tested in a phase 2 clinical trial in Malian children did not provide significant protection against all clinical malaria^21^. However, the vaccine demonstrated significant efficacy against clinical malaria caused by strains with the vaccine-type (3D7 strain) *ama1* sequence at the cluster 1 loop^21,22^.

Diversity in *ama1* consists of single nucleotide polymorphisms at more than 60 loci encoding amino acid changes throughout all three domains of the ectodomain, but concentrated in domain 1^9^. We previously identified 214 unique *ama1* ectodomain sequences among 506 single or predominant clone *P. falciparum* infections^9^ in Bandiagara, the town in central Mali where the AMA1 vaccine was tested in adults^23^ and children^21,24^. A prototype diversity-reflecting protein microarray that included 58 AMA1 variants showed increases in both lifetime and seasonal magnitude and breadth of anti-AMA1 antibodies in malaria-exposed Malian children and adults living in Bandiagara, where malaria transmission is intense but highly seasonal^25^. Here we report the use of an expanded protein microarray with 263 unique AMA1 variants isolated from the AMA1 vaccine trial site, as well as a high-density peptide array populated with overlapping 16-mer AMA1 peptides derived from our field isolates and publicly available sequences. Our aim was to examine the effects of both natural *P. falciparum* parasite exposure and AMA1 vaccination on the breadth and magnitude of strain-specific and epitope-specific antibody responses to AMA1.

## Results

### Study population

Sera were randomly selected from participants in a 3D7-based AMA1 vaccine trial conducted in Bandiagara, Mali to be probed on the whole protein microarray. Forty (40), AMA1-vaccinated children and 20 AMA1-vaccinated Malian adults with sera corresponding to pre-vaccination and 90 days post first AMA1 vaccination were selected. Additionally, 40 control-vaccinated Malian children and 20 control-vaccinated Malian adults were randomly selected, with sera corresponding pre-, peak, and post malaria season and probed on the whole-protein array. Sera from 11 North-American malaria-naïve blood donors were also probed on the whole protein array as malaria exposure negative controls. Sera from a subset of these participants were probed on the high-density peptide array, including 10 AMA1-vaccinated Malian children, 10 control-vaccinated Malian children, 10 control-vaccinated Malian adults, and five North American controls at the same vaccine and season-relative time points. Of the group of 10 AMA1-vaccinated Malian childrens’ samples run on the high-density peptide microarray, pre-and post AMA1-vaccination sera from six^6^ randomly-selected children were probed on a separate, mutation scan peptide microarray consisting of the hypervariable cluster 1 loop region.

### The magnitude of AMA1 seroreactivity and breadth of serorecognition increased with age

#### Whole-protein array

**Figure 1:**
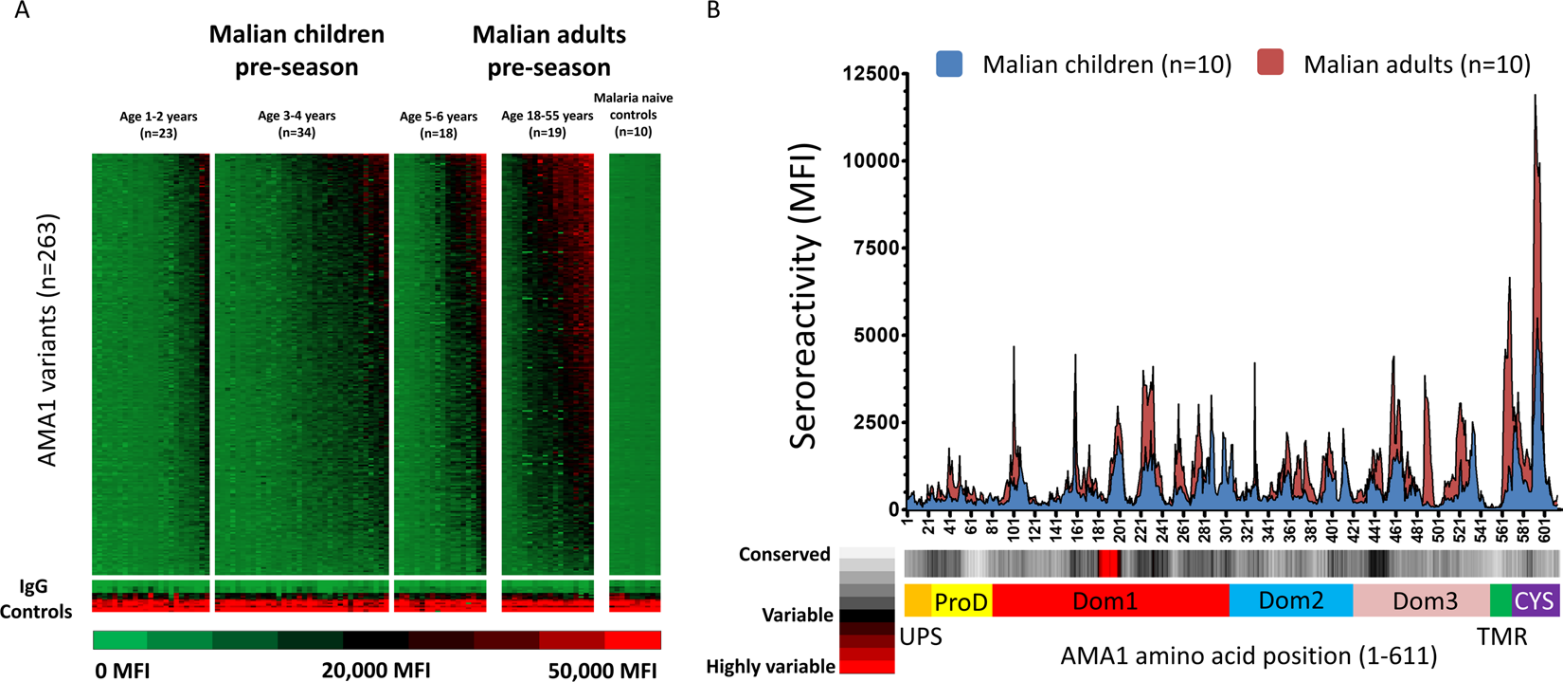
Magnitude of seroreactivity and breadth of serorecognition of AMA1 proteins and peptides increases with age. (**A**) Heat map of the pre-season seroreactivity to 263 AMA1 whole-protein variants and IgG positive controls (rows) in Malian children (age 1–6 years, n = 75), Malian adults (age 18–55 years, n = 19), and North American malaria-naïve controls (n = 10) in columns. Malian adults had higher median preseason seroreactivity to AMA1 variants than Malian children, and North American controls (p < 0.001, Mann Whitney test). Children ages 1–2 (n = 23) had lower seroreactivity to AMA1 variants than 3–4 (n = 34) and 5–6 (n = 18) year olds. (**B**) Mean pre-season seroreactivity to AMA1 peptides is greater in Malian adults than Malian children. Overlapping 16 amino acid AMA1 peptides are numbered by the first amino acid position. Malian pediatric (blue, n = 10) and adult (red, n = 10) seroreactivity was plotted along the length of the AMA1 sequence.

At the beginning of the malaria transmission season, the mean seroreactivity to the 263 AMA1 variants on the protein array was 81% higher in unvaccinated adults than unvaccinated children, and responses in both cohorts were greater than in North American malaria-naïve controls (*p* < 0.001, Mann-Whitney test, Fig. 1A and Supplementary Fig. S1A). The median percent serorecognition of AMA1 variants in unvaccinated Malian children and adults before the malaria season was 62% and 98%, respectively, with North American controls recognizing 6.8% of AMA1 variants above background (*p* < 0.001, Mann Whitney test, Supplementary Fig. S1B). The median pre-season seroreactivity to individual AMA1 variants was higher in children aged 3–4 and 5–6 years than in those aged 1–2 years (*p* < 0.05, *p* <  0.02, respectively, Mann Whitney test, Fig. 1A and Supplementary Fig. S1C). The breadth of serorecognition of AMA1 proteins was highly variable in Malian children six years and younger (standard deviation = 40.33%, Fig. Supplementary Fig. S1D).

#### High-density peptide array

Locations of residues defining domains and structural elements^7,26^ were as follows: Domain 1 (positions #83–303), the largest of the three domains, was represented by 4,150 unique 16mer peptides derived from available sequence data and was more polymorphic than domains 2 (positions #304–418) and 3 (positions #419–546). Domains 2 and 3 were represented by 1,507 and 1,707 peptides, respectively. Both immunoreactive epitopes and non-reactive regions were found across the full length of AMA1 (Fig. 1B). Seroreactive epitopes for unvaccinated Malian children and adults were detected in all three domains of the protein, with the highest magnitude of seroreactivity located outside the ectodomain in the cytosolic region (positions #567–611) (*p* < 0.05 for all tests, Mann Whitney test Fig. 1B and Supplementary Fig. S1E).

Sera from unvaccinated Malian adults had greater breadth of serorecognition compared to unvaccinated Malian children to all three AMA1 ectodomains and the intracellular cytosolic region individually (*p* < 0.001 for all tests, Mann Whitney test, Fig. Supplementary Fig. S1F), and overall (41.9% and 18.7% respectively, *p* < 0.001, Mann Whitney test).

### Breadth and magnitude of seroreactivity to AMA1 variants and specific AMA1 epitopes over the course of a malaria season

#### Whole-protein array

**Figure 2:**
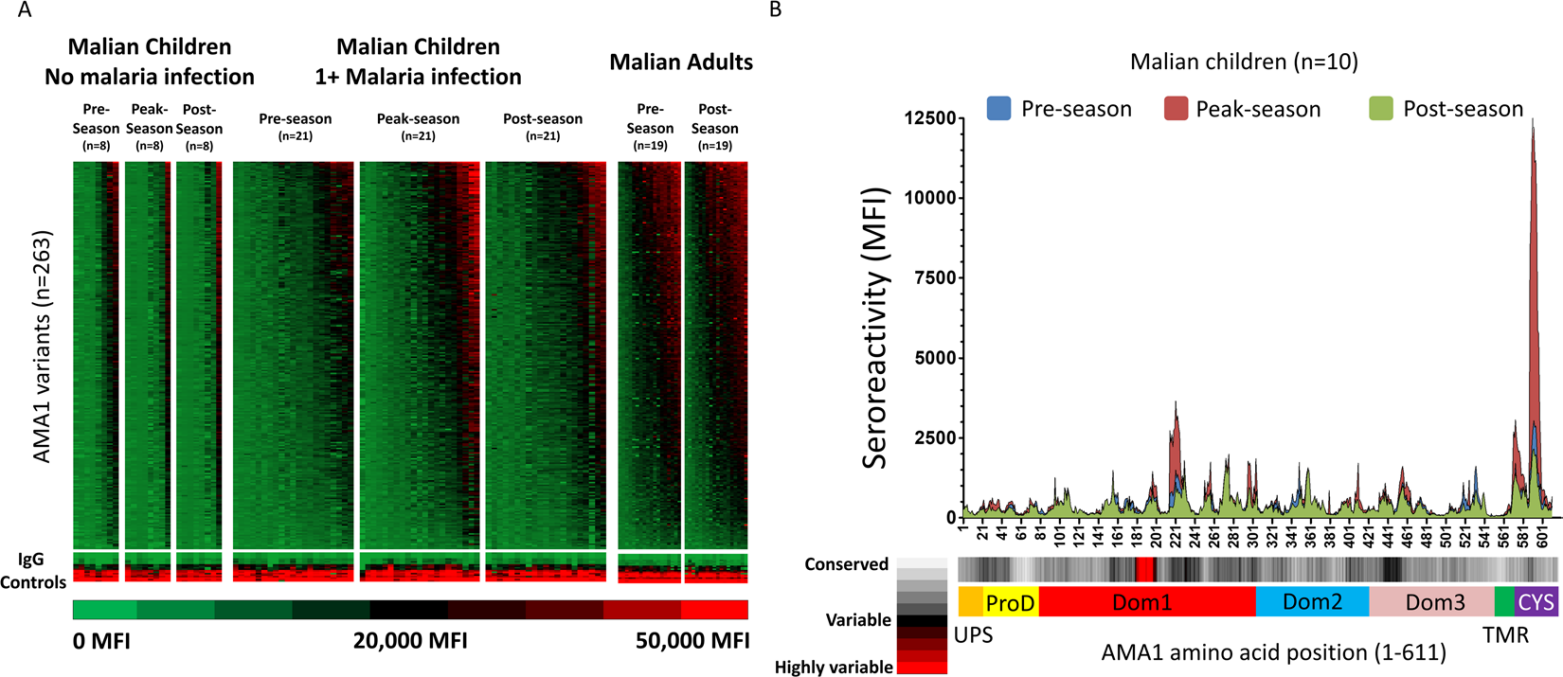
Magnitude and breadth of seroreactivity to AMA1 proteins and peptides increases over the malaria transmission season in children who had a clinical malaria episode. (**A**) Heat map of seroreactivity to 263 whole-protein AMA1 variants (rows) in Malian children who did (n = 21) and did not (n = 8) have a malaria positive sample during the malaria transmission season. Pre- (May/June 2007), peak- (September 2007) and post-season (December/January 2008/9) seroreactivity in Malian children and pre- (June 2005) and post-season (December 2005) seroreactivity in Malian adults is separated in columns. Malian children who had an infection had higher seroreactivity in the peak- and post-season than in the pre-season. Malian children who did not have an infection during the season and Malian adults did not differ in seroreactivity from pre- to post season. AMA1 variants are sorted from top to bottom by highest to lowest mean seroreactivity per group; individuals in each group separately are sorted from left to right by increasing mean seroreactivity. (**B**) Seroprofiles of mean seroreactivity to peptides show increases in seroreactivity to AMA1 linear epitopes in Malian children (n = 10) who experienced a clinical malaria illness during the malaria season from pre- (blue) to peak- (red) and post-season (green). Sera from Malian children reacted more strongly to peptides in the 1e-loop and the cytosolic region than other AMA1 peptides.

Twenty-one children in the control-vaccine group had a malaria infection detected by PCR or microscopy during the transmission season and had protein microarray data available for all seasonal cross-sectional time points (pre-, peak- and post-season). Eight children did not have a positive malaria blood sample during follow up, and had protein microarray data for all three time points. Sera from infected control-vaccinated Malian children showed significant increases in median magnitude of mean seroreactivity to 263 AMA1 proteins from pre- to peak-season, and pre- to post-season, but no difference from peak- to post-season (*p* = 0.001, *p* < 0.0001, *p* = 0.58 respectively, Wilcoxon sign-rank test, Fig. 2A and Supplementary Fig. S2A). Control-vaccinated Malian children and adults did not have a significantly increased breadth of serorecognition of AMA1 proteins measured over the course of the malaria season (*p* > 0.19 for all tests, Wilcoxon sign-rank test, Fig. 2A, Supplementary Fig. S2B).

#### High-density peptide array

A subset of 10 control-vaccinated children who had a malaria infection during the transmission season and had protein array data for all seasonal time points were randomly selected from those tested on the whole-protein array for study on the peptide array. This group had an increase in median seroreactivity to AMA1 peptides at the peak of the malaria season that decreased to preseason levels by the end of the malaria season. (*p* = 0.048 pre- to peak season, p = 0.084 peak- to post- season, *p* = 0.77 pre- to post- season, Wilcoxon signed-rank test, Fig. 2B and Supplementary Fig. S2C). Separating peptides by AMA1 domain revealed the median increase seen in all peptides from pre- to post- season was driven by increases in seroreactivity to peptides in domain 1 and very high increases in median seroreactivity to peptides in the cytosolic region. (Supplementary Fig. S2C). Seven of the ten control-vaccinated children had at least one malaria infection between pre- and peak- season cross-sectional time points. Breadth of serorecognition to AMA1 peptides increased from pre- to peak-season in all domains and the cytosolic region, and waned to pre-season levels by the end of the transmission season (Supplementary Fig. S2D).

### Reactivity to AMA1 protein and peptide variants after AMA1 vaccination

#### Whole-protein array

**Figure 3:**
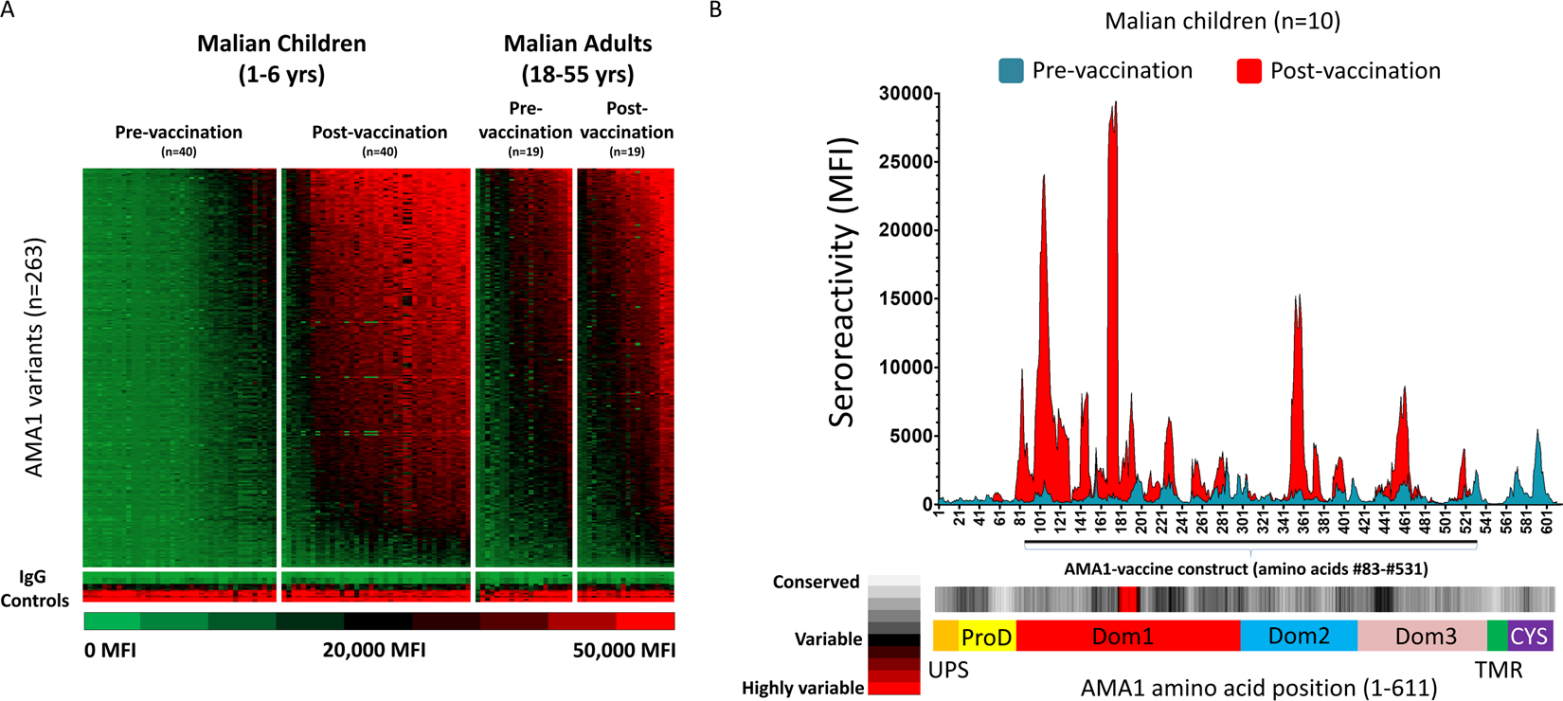
Sera from AMA1 vaccination react strongly to the majority AMA1 whole-protein variants and is biased towards four distinct epitopes. (**A**) Heat map of seroreactivity to 263 AMA1 protein variants and serially diluted IgG positive controls (rows) in AMA1 vaccinated Malian children (n = 40), and adults (n = 19) (columns) pre- and 90 days post-vaccination. Individuals are sorted from lowest to highest from left to right within each cohort. (**B**) Bar plot displaying the mean seroreactivity to AMA1 peptides by amino acid position in AMA1-vaccinated children, pre-vaccination (blue) and 90 days post-vaccination (red). AMA1 vaccine construct (amino acids #83-#531) shown with black bar. Antibodies that proliferated after AMA1 vaccination target four major epitopes, and no change is observed in peptides corresponding to regions outside of the AMA1 vaccine construct.

Both vaccinated Malian children and Malian adults had increased levels of anti-AMA1 antibodies 90 days after the first immunization with the monovalent, AMA1-based subunit vaccine FMP2.1/AS02_A_, compared to pre-vaccination, as measured by the magnitude of seroreactivity on the whole-protein array (Fig. 3A and Supplementary Fig. S3A,B). Median AMA1 seroreactivity increased 300% among AMA1-vaccinated children (*p* < 0.001), compared to an 11% increase in children in the control group during the same time period (*p* = 0.52, Wilcoxon sign-rank test, Fig. 3A, Supplementary Fig. S3C). On average, sera from AMA1-vaccinated children recognized 49.1% of AMA1 variants on the protein microarray before vaccination, compared to 96% of variants 90 days after AMA1 vaccination (*p* < 0.0001, Wilcoxon sign-rank test, Fig. Supplementary Fig. S3D). AMA1-vaccinated adults experienced a 31% mean increase in the magnitude of AMA1 seroreactivity during the first 90 days, compared to a statistically insignificant decrease in control-vaccinated adults in the same time period (*p* = 0.004, *p* = 0.22, Wilcoxon sign rank test, Supplementary Fig. S3C). Adults were seropositive for 95.7% of AMA1 variants before AMA1 vaccination, and serorecognition increased to 99.1% serorecognition 90 days post-vaccination (*p* = 0.09, Wilcoxon sign-rank test, Supplementary Fig. S3D). Reference AMA1 3D7 (AMA1-193.1) was not the most seroreactive variant after AMA1 vaccination, ranking 47^th^ and 29^th^ out of 263 variants in children and adults, respectively (Supplementary Fig. S3A,B).

#### High-density peptide array

**Figure 4:**
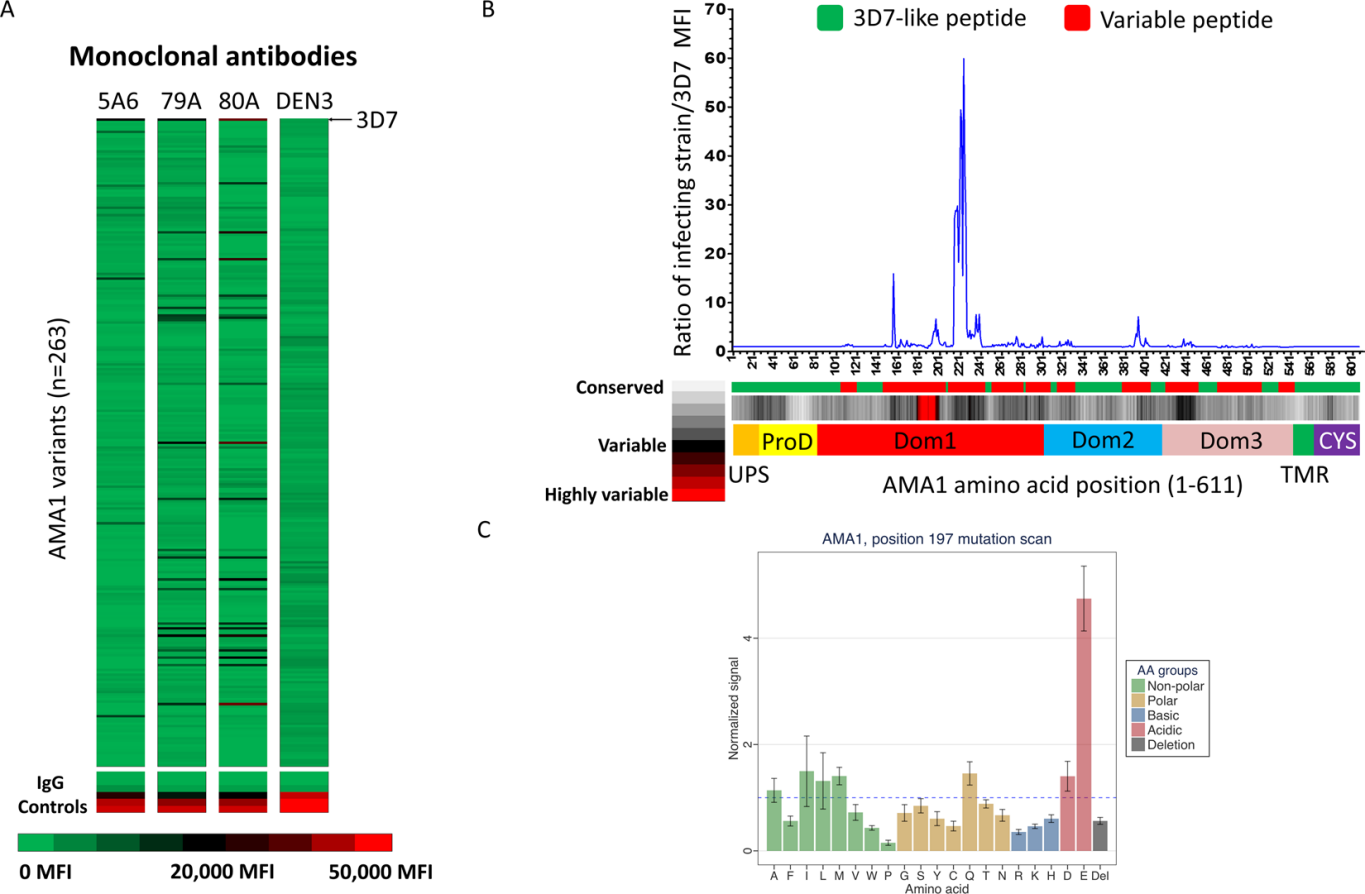
Seroreactivity to AMA1 whole-proteins and peptides is strain specific. (**A**) Mean seroreactivity (n = 4 per monoclonal antibody) of four monoclonal antibodies (columns) to 263 AMA1 variants and IgG-positive controls (rows). Monoclonal antibodies 5A6, 479 A and 480 A recognized 5, 10, and 15 AMA1 variants respectively. Dengue virus monoclonal antibody DEN3 did not recognize any AMA1 whole-protein variant. (**B**) Mean fold-difference in seroreactivity to peptides matching the infecting parasite sequence (compared to reference strain 3D7) by amino acid position in control-vaccinated Malian children (n = 7), who had a clinical malaria illness within 90 days after vaccination. Regions that were 100% conserved (green) among all of the infecting strains and 3D7, differed by at least one amino acid (red) are indicated below. (**C**) Single amino acid changes result in significant changes in seroreactivity to cluster 1 loop peptides in Malian children 90 days after AMA1 vaccination. Bar plot with mean normalized signal +/− standard error of seroreactivity among children (n = 6) 90 days post-vaccination with AMA1. Colors denote polarity of the amino acid substitution; non-polar (green), polar (yellow), basic (blue), acidic (red), and deletion of the amino acid at position 197 (black). Dashed blue line indicates a normalized signal of 1, the theoretical value where all mutations produce equal signal. Cluster 1 loop peptides containing vaccine-strain 3D7 glutamic acid at position 197 (E197) of the cluster 1 loop had the highest reactivity to AMA1-vaccinated Malian children.

In ten AMA1-vaccinated children the median seroreactivity to AMA1 peptides increased from baseline to 90 days after first vaccination (p < 0.002, Mann-Whitney test, Fig. 3B and Supplementary Fig. S3E). These children did not have a malaria infection during the first 90 days of follow-up. There was significantly increased seroreactivity to epitopes in domains 1, 2, and 3 following AMA1 vaccination among the 10 children whose sera were probed on the peptide array (p < 0.01 all tests, Wilcoxon sign-rank test, Supplementary Fig. S3E). The four most immunoreactive epitopes were in domain 1 (positions #96–120 and #166–180), domain 2 (amino acid #346–368) and domain 3 (positions #449–469) (Fig. 3B). Among AMA1-vaccinated children, 90 days after vaccination, there was no detectable change in seroreactivity to AMA1 peptides residing outside of the 449 amino acid vaccine construct (positions #83-#531) (Fig. 4B and Supplementary Fig. S3E). Breadth of serorecognition to AMA1 peptides increased in all three domains, but not the cytosolic region that lies outside of the vaccine construct (Supplementary Fig. S3F) (p < 0.01 for all tests, Wilcoxon sign-rank test).

### Strain specificity of mono- and polyclonal antibodies to diversity-reflecting AMA1 protein and peptides

#### Whole-protein array

We probed three AMA1 monoclonal antibodies (mAbs) and one control Dengue virus monoclonal antibody to demonstrate strain-specificity on the whole-protein microarray platform. Monoclonal antibody 5A6, a known strain-specific, malaria inhibitory antibody that binds to a strain-specific domain-2 epitope, bound significantly to five AMA1 variants on the array above Dengue virus mAb control. The magnitude of seroreactivity to AMA1 variants of mAb MRA-479A (N3-2D9) and MRA-480A (N3-1D7) were highly correlated (Fig. 4A and Supplementary Fig. S4, p < 0.0001, Pearson’s r = 0.89, r^2^ = 0.79). MAb 479 A is described as a <30% inhibitory antibody of malaria parasite FVO, and mAb 480 A has no known inhibitory activity. The exact binding locations of mAb 479 A and 480 A are not known. The binding loci for N3 negative control mAb DEN3; raised against dengue virus serotype 3, did not bind to AMA1 variants above empty-vector control features, yet bound to pan-IgG positive control features printed on the array (Fig. 4A). AMA1 variant 193.1 was identical to reference strain 3D7 at 62 polymorphic loci identified previously^9^ and had the highest monoclonal antibody reactivity on average of all AMA1 variants on the array (Fig. 4A).

#### High-density peptide array

Among the control-vaccinated children whose sera were probed on the high-density peptide array, seven experienced at least one clinical malaria episode between the pre- and peak-season serosurveys. The infecting malaria parasite sequence was determined for each infection. In total, 302 out of 611 peptides across the length of AMA1 were conserved among all infecting parasite strains and reference strain 3D7. Using peak season (post infection) sera for this subset of unvaccinated Malian children, the magnitude of seroreactivity to peptides matching the infecting strain(s) was compared to the seroreactivity to the peptide at the same locus that matched the reference strain 3D7 and reported as a ratio (Fig. 4B). Overall, the mean magnitude of seroreactivity of infecting strain peptides was 2.79 times higher than the seroreactivity of peptides matching reference strain 3D7 within the variable regions (red) among unvaccinated, Malian children who experienced at least one clinical malaria infection between pre- and peak season. The epitopes with the greatest fold differences between homologous and reference seroreactivity were located in domain 1, notably in peptides containing amino acids within loops surrounding the hydrophobic cleft; the cluster 1 loop (positions #197–211, 6.7-fold difference) and the 1e loop (positions #225–235, 59-fold difference) (Fig. 4B).

The Malian adults studied had been exposed over their lifetimes to many different *P. falciparum* parasites expressing diverse AMA1 variants. To examine the variability in seroreactivity to epitopes with differing amino acid content we plotted the minimum, median, and maximum mean seroreactivity to variant peptides located at the same amino acid positions across the length of the AMA1 sequence, for ten control-vaccinated Malian adult sera probed on the high-density peptide array (Supplementary Fig. S5A). There was high variability in seroreactivity with amino acid substitutions (minimum and median seroreactivity were 9% and 26% of maximum seroreactivity respectively). Additionally, the minimum, median, and maximum seroreactivity to peptides at a given locus varied substantially among AMA1-vaccinated children 90 days after vaccination (Supplementary Fig. S5B). The most diverse region of the AMA1 molecule, the cluster 1 loop, had very low median seroreactivity compared to the maximum at that epitope in both adults and children.

Pre- and post AMA1 vaccination sera from six children were analyzed with a mutation scan peptide microarray to evaluate the strain specificity of amino acids within the cluster 1 loop of AMA1 (amino acid positions 190–206). This subset of children were randomly selected from the subset of ten children whose sera were run on the peptide array. Six children were chosen due to the mutation scan array design, which allowed 12 samples (six pre- and six post-vaccination) to be probed on one slide, and no statistical power assessment was performed *a priori*. The greatest mean seroreactivity was seen with vaccine-strain glutamic acid (E) in position 197 (E197) with a mean normalized signal of 4.75 (Fig. 4C). Substitutions at this position resulted in dramatically reduced mean seroreactivity. Mutation scan analysis of pre-vaccination samples from the same individuals did not result in statistically significant changes in seroreactivity. These findings indicate that the antibody response after vaccination was specific to AMA1 protein with a glutamic acid at position 197, matching the vaccine strain. Of the 331 unique, field-derived (263) and publicly available (68) full-length AMA1 sequences used to populate the array, E197 was found in 11% of samples.

## Discussion

This study used diversity-reflecting protein and peptide microarrays to understand naturally-acquired and vaccine-induced antibody responses to a highly polymorphic malaria surface protein and leading vaccine antigen. Natural infection gave rise to both short-lived, broadly cross-reactive antibodies as well as strain-specific antibody responses targeting diverse epitopes previously associated with both natural and vaccine-induced clinical immunity. Contrary to expectation, vaccination with a monovalent blood-stage vaccine that elicited strain-specific efficacy was followed by extremely broad and strong recognition of hundreds of variants of the vaccine antigen, as well as increases in seroreactivity to four distinct epitopes.

Probing sera on the whole-protein microarray measured the binding of antibodies to tertiary structures with both conformational and discontinuous epitopes. A limitation of this platform is its inability to ensure proper folding of proteins expressed using a cell-free *E. coli*-based transcription/translation system, but this system has been validated as a means of measuring clinically relevant antibody responses^25,27–29^.

The observation that the whole protein variant 193.1, which was 100% identical to vaccine strain 3D7 at 62 polymorphic loci, was not the most seroreactive variant among AMA1-vaccinated children or adults, was surprising. This could be explained by imprecise folding of the protein as printed on the array, or variability in the concentration of antigen deposited on the array. All variants used in the printing of the arrays were obtained from the same cloning, expression and purification experiment, to rule-out within-protein batch effects. Antibody seroreactivity as measured on the whole-protein array represents the summation of the binding of a complex collection of polyclonal antibodies to both conserved and strain-specific epitopes, and is affected by recent and cumulative exposures to malaria parasites. It is possible that previous exposure to malarial parasite AMA1 could influence the strain-specific serological response to AMA1 vaccination. The protein array is unable to detect responses to individual regions of the molecule that are targeted preferentially in response to natural infection or AMA1 vaccination.

Seroprofiles generated by probing sera on the high-density linear peptide array provide measurements of the antibody responses to individual epitopes, and the resolution to investigate the effect of single amino acid substitutions on antibody seroreactivity to these polymorphic epitopes. Using this platform, we measured the relative immunogenicity of domains and individual epitopes to uncover immunodominant regions. The flexible linear epitopes on the peptide array undoubtedly fail to represent some important conformational and discontinuous epitopes. With short linear peptides, it is also possible that short amino acid sequences are shared between different organisms, and not specific to malaria. However, combining the two methodologies provided a broader picture of antibody responses in population-based studies, with each platform’s strengths compensating to some degree for the limitations of the other.

Clinical immunity to malaria is thought to be acquired only after an individual has been exposed to a variety of genetically distinct parasites^30,31^. Infection by different malaria strains may lead to the generation of diverse antibodies that prevent new clinical malaria episodes upon re-exposure to previously encountered variant parasite antigens. This model is supported by molecular epidemiological evidence showing that children experiencing the second of two consecutive infections with genotyped AMA1 variants had a greater likelihood of clinical symptoms when that second infection differed from the preceding one at key polymorphic loci (e.g. at domain 1, cluster 1 loop, amino acid 197)^9^. Supporting this model of clinical immunity to malaria, in the present study, Malian adults with lifelong exposure had higher magnitude and breadth of serorecognition to AMA1 variants than Malian children.

Surprisingly, the distribution of serorecognition in children was bimodal; children either recognized the majority of AMA1 whole-protein variants or just few. This phenomenon may be explained by high seroreactivity to the immunodominant cytosolic region of the AMA1 protein, which is more conserved. With each exposure to malaria parasites, seroreactivity to epitopes including those in the conserved cytosolic region may increase, eventually reaching a point at which children cross a threshold of seroreactivity to recognize the majority AMA1 whole-protein variants above background.

Comparing seroprofiles of adults to those of children using both platforms permitted inference of which targets of AMA1 antibodies are correlated with protection against clinical malaria, with the important caveat that it is difficult to distinguish markers of protection from markers of exposure. Adults had higher magnitude seroreactivity to structural loops surrounding the binding site of the AMA1-RON2 invasion complex. Adults also recognized more diverse variants of these loops than did children, and were able to mount effective multi-strain antibody responses to a larger collection of diverse malaria parasites. This observation could lead to a better serological correlate of protection from clinical malaria disease that is not subject to spurious results due to the strain-specificity of antibodies to malaria structural epitopes.

The initial sharp increase in AMA1 antibody titers as measured by ELISA using a reference antigen has been reported to wane within six weeks after a single malaria infection^32–35^. Our data show that diverse strain-specific antibody responses similarly wane quickly. We observed an increase in childrens’ antibody titers over the malaria season that peaked and then declined in the post-season as transmission ended. While the seasonal change in seroreactivity to AMA1 proteins was not significant for children overall, the subset of children who experienced a clinical malaria episode had a significant increase in AMA1 seroreactivity, with peak seroreactivity corresponding to the peak of the malaria transmission season.

Children had increased seroreactivity to all AMA1 whole-protein variants on the array after a clinical malaria episode, regardless of the strain of the infecting parasites. This may be explained by the large increase in seroreactivity to epitopes in the more conserved intracellular cytosolic component of the AMA1 molecule seen on the peptide array following clinical episodes. Antibodies targeting the 1e-loop in the hydrophobic cleft have demonstrated inhibitory activity^7^. A sharp increase in seroreactivity was observed in recent infections within the immunodominant cytosolic region of AMA1 that waned with time. Studying the dynamics of seroreactivity to specific peptides could potentially yield sensitive serological biomarkers for malaria exposure within discrete time frames in the late stages of malaria elimination.

The FMP2.1/AS02_A_ vaccine contains the ectodomain of only the laboratory reference strain 3D7 AMA1, and we therefore expected the magnitude of seroreactivity to the vaccine strain 3D7 AMA1 variant to be significantly higher than seroreactivity to other whole-protein AMA1 variants after vaccination. However, both children and adults had a broad increase in AMA1 seroreactivity on the whole-protein microarray following vaccination. Clinical protection was observed only when individuals were infected with a parasites homologous to the vaccine strain with respect to the protein sequence of the cluster 1 loop^21^. While there was significant variability in the magnitude of seroreactivity to different variants of AMA1 proteins, common protein motifs that correlated with vaccination or protection could not be identified. This may be due to the complexity of the polyclonal antibody response as measured on the whole protein array, with hundreds of competing antibodies binding to both variable and conserved regions of the molecule.

Peptide array analysis of sera from AMA1-vaccinated Malian children revealed the presence of at least four immunodominant epitopes, each with variable levels of amino acid conservation. The sum of seroreactivity to peptides within these four epitopes alone far eclipsed the seroreactivity to every other AMA1 peptide combined. This would explain the broad reactivity observed on the protein array: seroreactivity to any one of these four epitopes would obfuscate any sequence-dependent differences in antibody reactivity that may exist. Without these insights from the peptide array, probing sera from AMA1-vaccinated children on the whole-protein array alone would lead to the false conclusion that the vaccine had strain-transcending immunogenicity.

While a protective vaccine does not necessarily need to generate antibodies mimicking those elicited by natural infection, it may be important to note the differences between seroprofiles in clinically immune individuals and those seen following vaccination. Our results showed that antibodies circulating in clinically immune Malian adults after a lifetime of *P. falciparum* infection differed from those measured after AMA1 vaccination in Malian children in the epitopes they preferentially targeted, likely in part because the vaccine was comprised of the AMA1 ectodomain without including the cytosolic region.

Despite the clear evidence from previous studies of strain-specific natural and vaccine-induced immunity to AMA1, strain-specificity of the antibodies generated after malaria infection and AMA1 vaccination could not be demonstrated using the whole-protein array. As noted above, this inability may be explained, in part, by the immunodominance and relative conservation of the cytosolic region. Antibodies targeting epitopes in the cytosolic region may overwhelm any detectable seroreactivity to epitopes displaying antigenic variation in domains 1–3. Conversely, probing monoclonal antibodies on the whole-protein microarray demonstrated the platform’s ability to analyze the breadth of cross-reactive inhibitory antibodies, which may facilitate discovery of protective epitopes and inform vaccine development. Notably, the strain-specificity of antibody responses to individual peptide variants was observed at the same loci among different individuals. These commonly recognized loci targeted by strain-specific antibodies included polymorphic structural loops previously implicated in natural and vaccine-induced allele-specific immunity as well as inhibitory monoclonal antibodies^21,36,37^.

Among clinically immune Malian adults, there remained significant “lacunae” in their serorecognition of field-derived variant malaria peptides located in immunogenic epitopes. This first analysis was not designed to detect a minimum threshold of seroreactivity to epitopes needed to protect against clinical disease. Prospective longitudinal seroepidemiological studies measuring the association of seroreactivity to immunogenic epitopes and the risks of clinical malaria illness with homologous and heterologous parasites could demonstrate the basis for strain-specific clinical immunity. To facilitate this, next-generation diversity-reflecting protein and peptide microarrays will contain more variants of more antigens.

Effective malaria vaccines must do more than just elicit a strong immune response—the immune response must effectively inhibit parasite survival and replication, and ideally, the response should be strain-transcending^38^. Our earlier studies demonstrated that the AMA1 vaccine decreased the incidence of clinical malaria caused by parasites homologous to the vaccine strain with respect to the cluster 1 loop. Using a mutation scan method, we pinpointed a single amino acid (aa197) as a key determinant of the magnitude of strain-specific antibody responses to cluster 1 loop peptides after AMA1 vaccination. The relative importance of hypervariable locus position 197 was calculated using machine-learning random forest analysis^9^, and relative risk ratio models^22^, and was measured directly in this analysis. The effectiveness of a malaria vaccine displaying significant strain-specific efficacy would be limited by simultaneous or sequential infection with multiple parasite clones, which is common^39–41^.

Protein and peptide arrays populated with field-derived variants of vaccine proteins are powerful tools to assess the immunogenicity of vaccine constructs and the strain-specificity of antibodies generated by vaccination. Peptide arrays are more expensive, and the analysis is labor-intensive, limiting their feasibility for routine sero-surveillance. Whole-protein arrays are relatively robust and inexpensive, and results can be reported within hours. While protein arrays are being developed for use in the field, like ELISA, they cannot easily characterize strain-specific or polyclonal antibody responses. The translation of these platforms into practical tools for seroepidemiology will require the thoughtful use of informative, strain-transcending biomarkers and economies of scale. Vaccine research may benefit from employing high-resolution peptide microarrays early in development to assess the strain-specificity of functional monoclonal antibodies and the serological responses to vaccination well before vaccines against malaria or other pathogens that use genetic diversity to evade the immune system are taken to the field for testing in humans.

## Methods

### Study site

Bandiagara is a rural town with a population of approximately 14,000 inhabitants located in an area of seasonal malaria transmission in Mali. *P. falciparum* malaria transmission coincides with the rainy season between June and November. Entomological inoculation rates (EIR) range from a peak of 60 infected mosquito bites per person per month in September, to virtually no transmission during the dry season (December to May). The total annual entomological inoculation rate per person is estimated to be from 50 to 150 infected bites^42^. The vast majority (97%) of malaria infections are due to *P. falciparum*, with rare *P. malariae*, and *P. ovale* infections. Children under six years of age vary in their risk of clinical malaria episodes, ranging from none to more than four clinical episodes of malaria per year, depending on neighborhood of residence^42,43^.

### Serum samples

Sera were selected from two completed clinical trials of FMP2.1/AS02_A_, conducted in Bandiagara^21,23^. FMP2.1/AS02_A_ is an AMA1-based vaccine consisting of the 449 amino acid AMA1 ectodomain (amino acids #83–531) of *P. falciparum* strain 3D7 (Walter Reed Army Institute of Research) and formulated with the proprietary AS02_A_ adjuvant (GlaxoSmithKline Biologicals)^44,45^. In both studies, a licensed rabies vaccine (RabAvert) was administered to control groups.

Adult serum samples were collected from Malians aged 18–55 years who had participated in a previously described phase I trial conducted from 2004 to 2005 in Bandiagara^23^. Participants who received the full dose of either the FMP2.1/AS02_A_ (n = 20) or rabies control vaccine (n = 18) and had samples for all study time points were included in this study. Pre-vaccination sera were collected on the day of the first of three vaccinations, at the end of the malaria season (December, 2004). Sera were also collected and analyzed 90 days after the first vaccination in March 2005, when peak AMA1 antibody titers measured by IgG ELISA were observed in the AMA1-vaccinated cohort^23^. In addition, sera from the rabies-vaccinated adult cohort from the beginning (June 2005) and end (December 2005) of the malaria transmission season were probed on the array to detect differences in antibody reactivity after one malaria season. A subset of 10 control-vaccinated adults was randomly selected from the original 19 samples at the May time point and probed on the high-density peptide array.

Pediatric serum samples were collected from children aged one to six years who participated in a previously-described phase II AMA1 vaccine (FMP2.1/AS02_A_) trial conducted in Bandiagara, Mali from 2007–2009^21^. Children were vaccinated at the beginning of the malaria transmission season, May/June 2007; and peak AMA1 antibody titers were observed 90 days after first inoculation in September 2007. Post-season samples were collected from December 2007 through January 2008. Sera from 40 children in the AMA1 vaccine arm and 40 children in the rabies control vaccine arm were selected by random number generator (R Bioconductor) from the 200 originally randomized to each experimental arm in the vaccine trial.

Previously collected sera from 11 North-American malaria-naïve blood donors were randomly selected as malaria-naïve controls and run on the AMA1 whole-protein microarray. One North-American malaria-naïve control sample had high seroreactivity to malaria proteins, and was removed from the analysis as a statistical outlier.

### Ethical approval and informed consent

The study protocols were approved by institutional review boards of the Faculty of Medicine, Pharmacy and Dentistry, Bamako, Mali and the University of Maryland, Baltimore. Written informed consent was obtained from all study participants or their parents or guardians. All methods were performed in accordance with the relevant guidelines and regulations.

### Design and probing of the whole-protein microarray

Dried blood spots were collected during the phase II FMP2.1/AS02_A_ vaccine trial from both routine monthly clinic visits, and unscheduled sick visits. Samples from microscopically-detectable *P. falciparum* infections underwent DNA extraction and PCR amplification of *ama1*^21^. Following capillary sequencing, Sequencher software (Genecodes) was used to identify single and predominant clone infections, and unique *ama1* haplotypes were then amplified using a specific primer-linker sequence that facilitated cloning into a pXI plasmid vector. Cloning, transformation, and amplification protocols have been described^46^. Single-clone *ama1* haplotype translation was ensured by use of a single colony amplification protocol^25^. Two hundred and sixty-three unique *ama1* whole-protein sequences were observed among 412 sequences analyzed. Protein microarray printing, probing, data generation, and quality control protocols have been described^46^.

### Design and probing of the high-density peptide array

Three hundred and thirty-one full-length AMA1 sequences were derived from field samples (263 sequences) and publicly available sequences (68 sequences) and used to generate 16 amino acid long peptides which overlapped by 15 amino acids tiled across the length of AMA1^7,26^. Conserved peptide sequences were printed only once on the array, with every fourth AMA1 peptide sequence printed in quadruplicate for quality control purposes. High-density peptide synthesis, sera probing and imaging protocols are described in Supplemental Methods.

The peptide array was used to probe sera from 20 children selected from among those run on the whole-protein array. Ten AMA1-vaccinated children selected to be run on the peptide array were randomly selected from samples that met the following criteria: the participant did not have a malaria-positive sample between the date of first vaccination and 90 days post vaccination, they were also run on the whole-protein AMA1 array, and also had a statistically significant increase from pre-vaccination in AMA1 antibody titer in response to the vaccine as measured by ELISA and the AMA1 protein microarray. Ten control-vaccinated children were randomly selected from among those who had at least one dried blood spot positive for malaria during the first malaria transmission season, with parasites sequenced for *ama1*. For each child, sera from pre-season, 90 days post-vaccination, and post-season time points were probed on the peptide array. Ten Malian adults and five North American adults were selected from among those run on the whole-protein array by random number generator (R Bioconductor) to be run on the array as well.

### Design and probing of the cluster 1 loop mutation scan peptide microarray

An additional peptide array was created to perform a pilot mutation scan analysis on the cluster 1 loop (c1L): 3D7 AMA1 positions 190–206 (MSPMTLDEMRHFYKDNK), to assess the strain specificity of the serological response after AMA1-vaccination in the hypervariable c1L region. The array consisted of novel peptides fabricated such that each position in the 17 amino-acid-long c1L sequence was changed to each of the 20 canonical amino acids or a deletion, yielding 357 unique peptide sequences. Each unique sequence was replicated 7 times on the array. Among the children selected for the peptide array analysis, pre- and post-vaccination sera from six randomly-selected AMA1-vaccinated children were probed on the cluster 1 loop mutation scan array. These children did not have a malaria-positive blood sample within the first 90 days of follow-up to ensure responses were due to the AMA1 vaccine and not natural exposure to malaria parasites. Six children were selected due to the limited capacity of the array and the pilot nature of the experiment.

### Monoclonal antibodies

Monoclonal antibody 5A6 was generated by, and ordered from the Walter Reed Army Institute of Research. Monoclonal antibodies MRA-479A and MRA-480A were obtained from Malaria Research and Reference Reagent Resource Center (MR4). Protocols detailing monoclonal antibody 5A6 preparation and characterization were previously described^7^. Information regarding mAbs 479 A and 480 A obtained by MR4 is available online at https://www.mr4.org/. Monoclonal antibodies were probed at 1 mg/mL concentration and quantitated according to standard protein array protocols^47^.

### Data analysis: signal preprocessing

The magnitude of seroreactivity to whole-protein AMA1 variants was calculated by scaling the raw median fluorescence intensity (MFI) of all probes on the protein array to 15 empty vector i*n-vitro* transcription/translation (IVTT) negative controls to adjust for differences in seroreactivity to the IVTT reaction buffers and cell-free *E. coli* machinery. For peptides, raw MFI represented the magnitude of peptide seroreactivity. AMA1 peptides were synthesized *in silico* without an IVTT reaction and no adjustment for background seroreactivity was necessary. The mean seroreactivity to AMA1 domains and structural loops was calculated by taking the mean seroreactivity to 16-mer peptides whose N-terminal amino acid, (the end unlinked to the substrate linker sequence) falls within the range identified as part of each structure or domain.

### Statistical analysis

All matched-pair analyses were done using the Wilcoxon sign-rank test. Group-wise, non-matched comparisons were conducted using the Mann Whitney test. Kruskal-Wallis non-parametric ANOVA was used to compare more than two groups. All statistical analyses were performed with R Project for Statistical Computing, Version 3.2.3.

### Strain-specific serological profiles

Strain-specific serological profiles were generated by matching 16-mer peptides to the sequence of the infecting parasite (homologous) and the reference strain 3D7 on which the vaccine is based. Seroreactivity to peptides homologous to the infecting parasite strain was then averaged by amino acid position among all children who had a clinical malaria episode during the first 90 days of follow up. Seroreactivity to peptides matching strain 3D7 was also averaged as a reference for comparison.

### Mutation scan data analysis

Normalized signal was calculated for each sample by amino acid position such that if all mutations at a position produced equal signal, the normalized signal for each mutation at that position will equal a value of 1. Normalized signal was calculated by taking signal *S* for sample *i* at position *j*, mutation *k*:

$${\rm{Normalized}}\,{{\rm{signal}}}_{{\rm{ijk}}}=\frac{{S}_{ijk}}{({\sum }^{}{S}_{ij})/21}$$

A neutral amino acid position, as defined by the substitution of all 20 potential amino acids and a deletion while still resulting in an equal signal with respect to the original amino acid, will yield a normalized signal of 1for all mutant sequences at that position. T-tests were performed at each position for each mutation to determine whether the normalized signals in the sample cohort were significantly greater than 1 after vaccination. Benjamini-Hochberg adjusted p-values were calculated resulting in 23 individual mutations, where association was reported with an adjusted p-value less than 0.05. Statistical analysis was performed in R version 3.0.2. T-tests were performed with: stats::t.test(data, mu = 1, alternative = “greater”). False discovery rate adjusted p-values^48^ were calculated with: stats::p.adjust(p.values, method = “fdr”).

## Supplementary information

Supplementary information.

Supplementary Data file S1.

Supplementary Data file S2.

## Data Availability

The datasets generated during and/or analysed during the current study are available from the corresponding author on reasonable request.
