# Supplementary material for: Microarray analyses reveal strain-specific antibody responses to Plasmodium falciparum apical membrane antigen 1 variants following natural infection and vaccination
Source: Sci Rep. 2020 Mar 3;10:3952. doi: 10.1038/s41598-020-60551-z (PMC7054363; doi:10.1038/s41598-020-60551-z)
Supplement: Supplementary file 1 — Supplementary information. [file 41598_2020_60551_MOESM1_ESM.pdf]

Supplementary Information

Microarray analyses reveal strain-specific antibody responses to *Plasmodium falciparum* apical membrane antigen 1 variants following natural infection and vaccination

Jason A. Bailey[1], Andrea A. Berry[1], Mark A. Travassos[1], Amed Ouattara[1], Sarah Boudova[1], Emmanuel Y. Dotsey[2], Andrew Pike[1], Christopher G. Jacob[3], Matthew Adams[1], John C. Tan[4], Ryan M. Bannen[4], Jigar J. Patel[4], Jozelyn Pablo[2], Rie Nakajima[2], Algis Jasinskas[2], Sheetij Dutta[5], Shannon Takala-Harrison[1], Kirsten E. Lyke[1], Matthew B. Laurens[1], Amadou Niangaly[6], Drissa Coulibaly[6], Bourema Kouriba[6], Ogobara K. Doumbo[6], Mahamadou A. Thera[6], Philip L. Felgner[2], and Christopher V. Plowe[1,7*]

[1]Center for Vaccine Development and Global Health, University of Maryland School of Medicine, Baltimore, MD

[2]Department of Physiology & Biophysics, University of California, Irvine, CA

[3]Wellcome Trust Sanger Institute, Hinxton, United Kingdom.

[4]Previous: Roche Sequencing Solutions, Madison, WI; Current: Nimble Therapeutics, Madison, WI

[5]U.S. Military Malaria Vaccine Program, Walter Reed Army Institute of Research, Silver Spring, MD

[6]Malaria Research and Training Center, University of Sciences, Techniques and Technologies of Bamako, Bamako, Mali

[7]Duke Global Health Institute, Duke University, Durham, NC

[*]Corresponding Author

Supplemental Materials and Methods

High-density peptide microarray synthesis.

Microarrays were synthesized with a Roche-NimbleGen Maskless Array Synthesizer by light-directed solid-phase peptide synthesis using an amino-functionalized plastic support (Greiner Bio-One) coupled with a 6-aminohexanoic acid linker and amino acid derivatives carrying a photosensitive 2-(2-nitrophenyl) propyloxycarbonyl (NPPOC) protection group (Orgentis Chemicals). Amino acids (final concentration 20 mM) were pre-mixed for 10 minutes in N,N-Dimethylformamide (DMF, Sigma Aldrich) with N,N,N',N'-Tetramethyl-O-(1H-benzotriazol-1-yl)uranium-hexafluorophosphate (HBTU, Protein Technologies, Inc.; final concentration 20 mM) as an activator, 6-Chloro-1-hydroxybenzotriazole (6-Cl-HOBt, Protein Technologies, Inc.; final concentration 20 mM) to suppress racemization, and N,N-Diisopropylethylamine (DIPEA, Sigma Aldrich; final concentration 31mM) as a base. Activated amino acids were then coupled to the array surface for three minutes. Following each coupling step, the microarray was washed with N-methyl-2-pyrrolidone (NMP, VWR International), and site-specific cleavage of the NPPOC protection group was accomplished by irradiation of an image created by a Digital Micro-Mirror Device (Texas Instruments, SXGA+ resolution), projecting 365 nm light. Coupling cycles were repeated to synthesize the full *in silico*-generated peptide library.

High-density peptide array sample binding and detection.

Prior to sample binding, final removal of side-chain protecting groups was performed in 95% trifluoroacetic acid (Sigma Aldrich), 0.5% Triispropylsilane (TIPS, TCI Chemicals) for 30 minutes. Arrays were incubated twice in methanol for 30 seconds and rinsed four times with reagent-grade water (Ricca Chemical Co.). Arrays were washed for one minute in TBST (1× TBS, 0.05% Tween-20), washed twice for one minute in TBS, and washed a final time for 30 seconds in reagent-grade water.

Serum samples were diluted 1:100 in binding buffer (0.01M Tris-Cl, pH 7.4, 1% alkali-soluble casein, 0.05% Tween-20) and incubated on arrays overnight at 4°C. After sample binding, the arrays were washed three times in wash buffer (1× TBS, 0.05% Tween-20), 10 minutes per wash. Alexa Fluor® 647-conjugated goat anti-human IgG antibody (Jackson ImmunoResearch) was diluted 1:10,000 (final concentration 0.1 ng/µl) in

secondary binding buffer (1x TBS, 1% alkali-soluble casein, 0.05% Tween-20). Arrays were incubated with secondary antibody for three hours at room temperature, then washed three times in wash buffer (10 minutes per wash) and once for 30 seconds in reagent-grade water. Fluorescence was detected by scanning at 635 nm at 2 µm resolution and 25% gain, using an MS200 microarray scanner (Roche NimbleGen).

Definitions

Pre-season. The beginning of the malaria transmission season in Bandiagara, Mali. May to June, 2007 for the pediatric cohort, and May to June 2005 for the adult cohort.

Peak-season. The height of the malaria transmission season, September 2007.

Post-season. The end of the malaria transmission season, December to January 2007/2008 for the pediatric cohort, and December to January 2005/2006 for the adult cohort.

Seroreactivity. A continuous variable that describes the amount of antibodies bound to a protein or peptide displayed as the median fluorescent intensity (MFI) generated by an array scanner.

Seropositivity. A dichotomous variable describing whether the MFI of an AMA1 protein variant is greater than three standard deviations above the median of empty-vector negative control. Protein array negative control probes consist of the *in vitro* transcription/translation machinery used when producing the proteins that lack the AMA1 sequence insert in the plasmid vector.

An individual was considered seropositive for an AMA1 peptide if the MFI for that peptide was at least three standard deviations above the mean of five malaria-naïve North American controls for the same peptide.

Breadth of serorecognition. The percent of AMA1 proteins and peptides that were seropositive out of the number of proteins and peptides on the array. This statistic is reported as percent seropositivity.

Broadly reactive. The ability of serum antibodies to bind to multiple variant AMA1 proteins or peptides at the same amino acid position.

Clinical malaria. Any treatment-seeking, or routine clinical visit where a patient has an axillary temperature greater than 37.5 °C, and more than 2,500 malaria parasites per µL of blood.

Domain definitions. Domains and structural elements were defined by amino acid positions previously reported (*7*). Peptides attributed to each domain and structural element correspond to peptides in which the first amino acid of the 16-mer peptide fall within the amino acid position described below. Peptides are counted towards two different regions if the first amino acid of a subsequent region is within 15 amino acids of the last amino acid in the previous region.

Signal peptide. Amino acids #1-#25.

Prodomain sequence. Amino acids #26-#82.

Domain 1. Amino acids #83-#303.

Domain 2. Amino acids #304-#418.

Domain 3. Amino acids #419-#546

Transmembrane region. Amino acids #547-#566.

Cytosolic region. Amino acids #567-#611.

1a-loop. Amino acids #135-#141.

1b-loop. Amino acids #170-#176.

1c-loop. Amino acids #184-#189.

1d/cluster 1 loop. Amino acids #196-#211.

1e-loop. Amino acids #225-#235.

1f-loop. Amino acids #263-#273.

Loop 2. Amino acids #347-#393.

Loop 3. Amino acids #456-#473.

Supplementary Fig. S1.

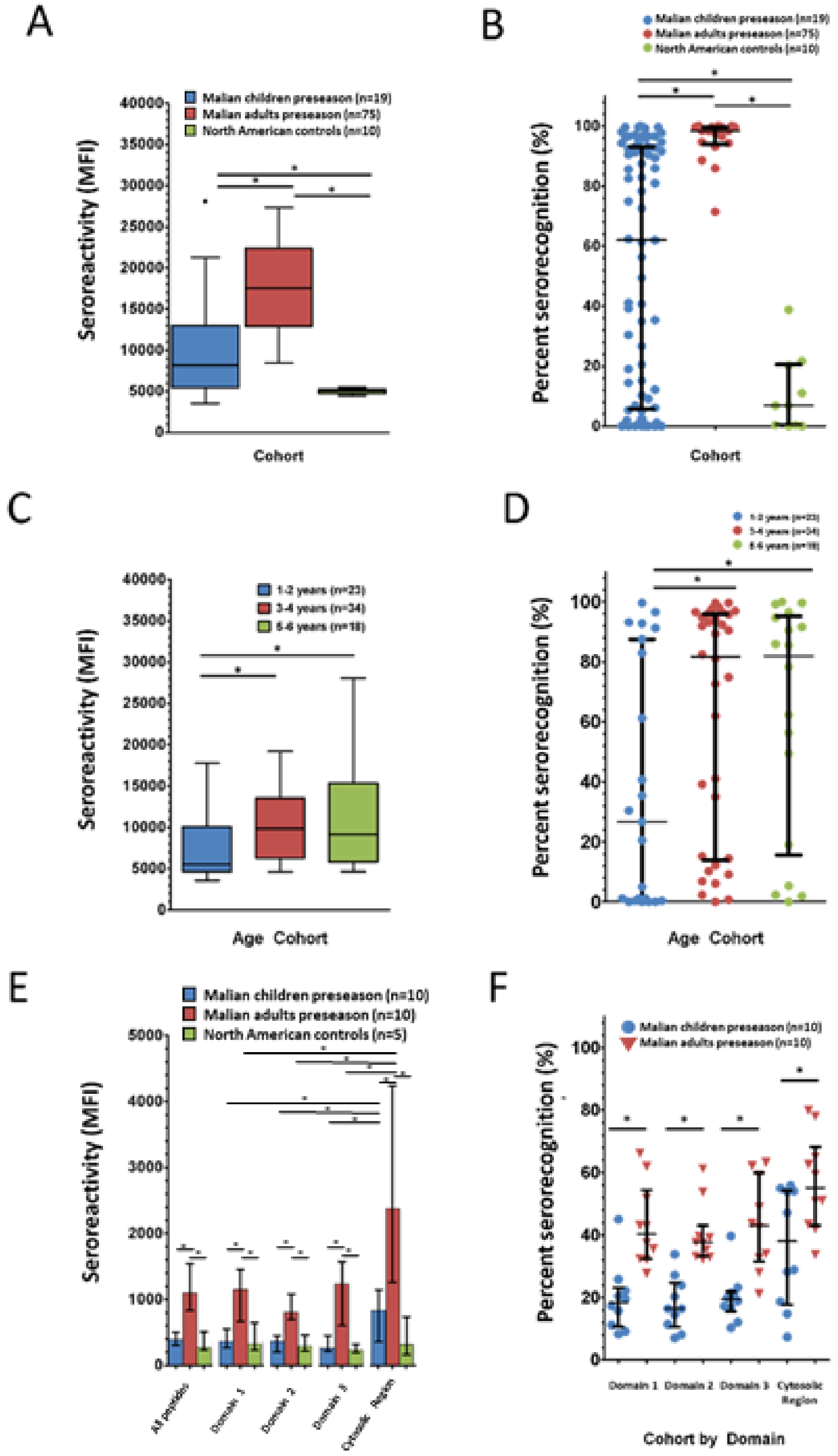

Supplementary Fig. S1. Seroreactivity to AMA1 whole-protein and peptide variants is higher in Malian adults than Malian children and North American controls and increases with age. a) Box and whisker plots show median and interquartile range of mean seroreactivity to 263 AMA1 variant proteins among Malian adults (n=19, red), Malian children (n=75, blue), and North American malaria-naïve volunteers (n=10, green). (*p<0.001, Mann-Whitney test) b) Dot plots display the pre-season percent serorecognition of AMA1 variants detected above 11 empty-vector IVTT controls in Malian children (n=75, blue), Malian adults (n=19, red) and North American controls (n=10, green). (*p<0.001 Mann-Whitney test) c) Box and whisker plots show of the median and interquartile range of mean pediatric pre-season seroreactivity to 263 AMA1 whole-protein variants by 2-year age cohorts; Malian children age 1-2 years (blue, n=23), 3-4 years (red, n=34), and 5-6 years (green, n=18). (* p<0.05 by Mann-Whitney test) d) Dot plots show the pre-season median and interquartile range of the percent serorecognition of 263 AMA1 whole-protein variants in Malian children age 1-2 (n=23, blue), 3-4 (n=34, red) and 5-6 (n=18, green) years. (*p<0.05 by Mann-Whitney test) e) Bar plots show median and interquartile range of pre-season seroreactivity to AMA1 peptides separated by domain in Malian children (blue, n=10), adults (red, n=10), and North American controls (green, n=5). f) Dot plots show the median and interquartile range of pre-season percent serorecognition of AMA1 peptides in Malian children (n=10, blue) and adults (n=10, red) separated by domain. (*p<0.001, Mann-Whitney test)

Supplementary Fig. S2.

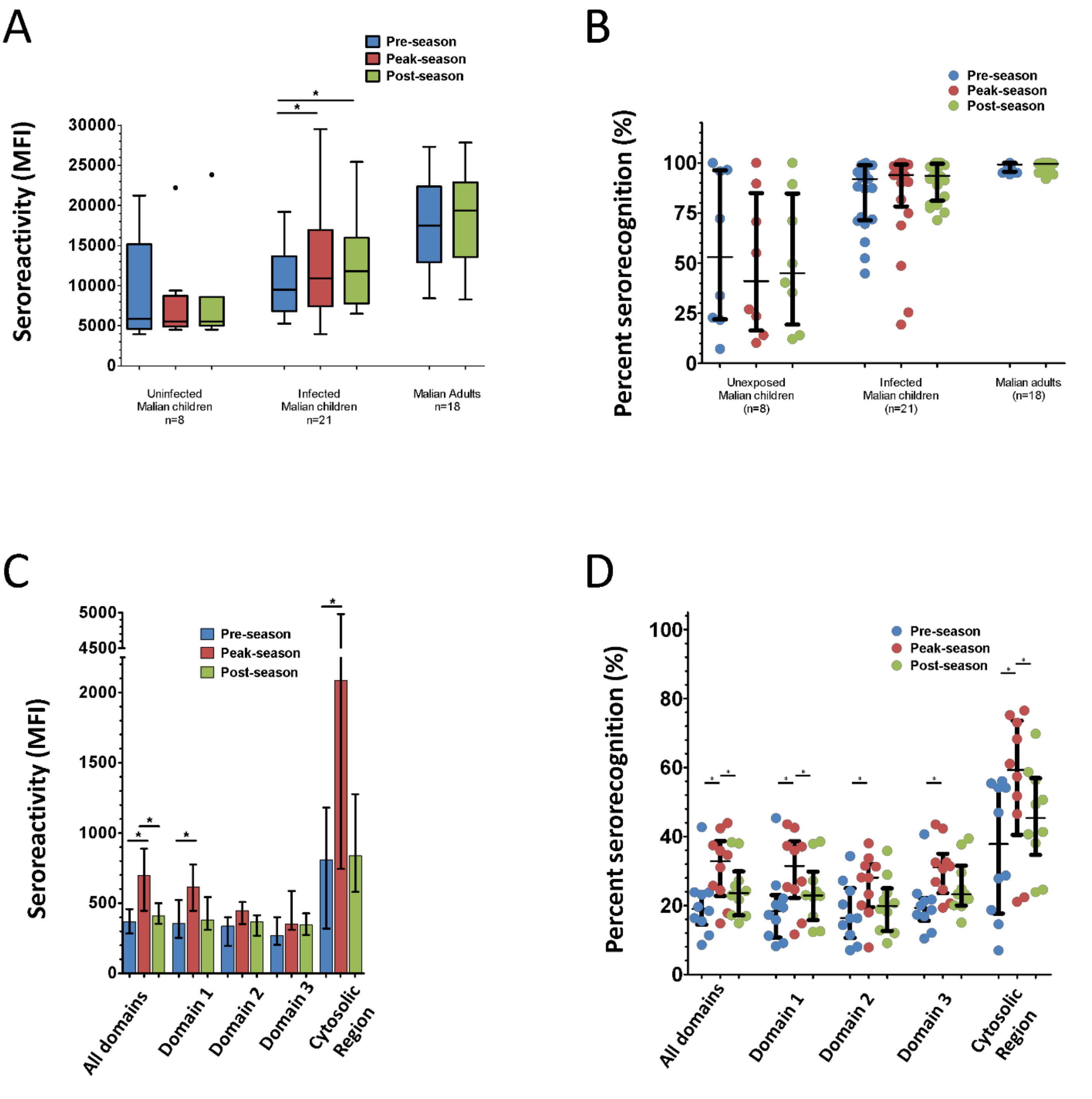

Supplementary Fig. S2. Seroreactivity and serorecognition of AMA1 proteins and peptides increases with malaria infection in Malian children. a) Box and whisker plots show the median and interquartile range of seroreactivity to AMA1 proteins in uninfected (n=8), infected (n=21), Malian children and adults (n=18) (*p<0.05, Wilcoxon signed-rank test). b) Dot plots show the median and interquartile range of percent serorecognition of 263 AMA1 proteins in infected and uninfected Malian children and Malian adults by season. c) Bar plots display the median and interquartile range of seroreactivity to AMA1 peptides separated by domain and colored by season in Malian children (n=10) who had a malaria infection during the transmission season (*p<0.05, Wilcoxon signed-rank test). d) Dot plots show median and interquartile range of percent serorecognition of AMA1 peptides separated by domain and season (*p<0.05, Wilcoxon signed-rank test). Seasonal cross-sectional time points are labeled pre- (blue), peak- (red), and post-season (green) for all cohorts.

Supplementary Fig. S3.

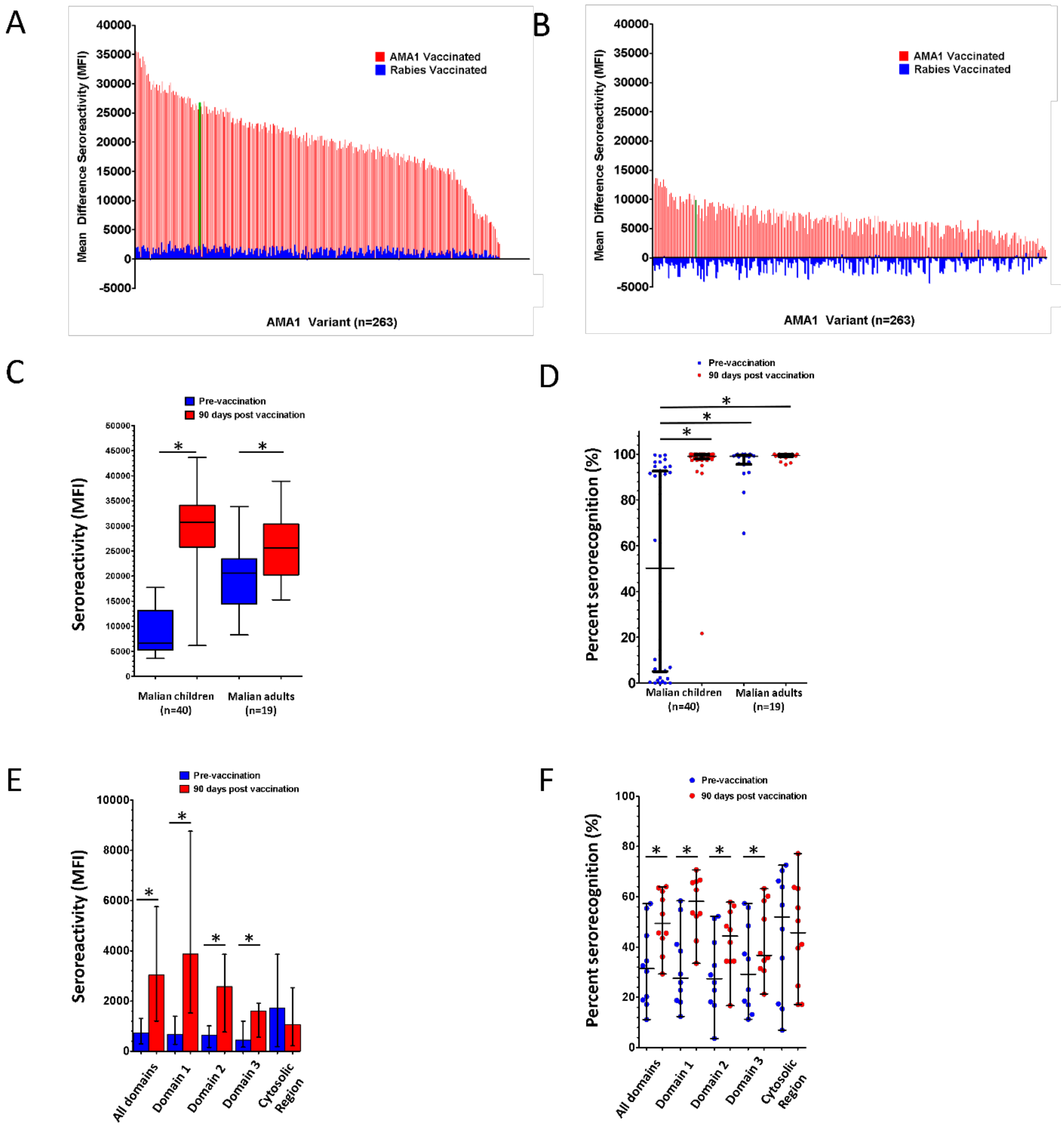

Supplementary Fig. S3. Seroreactivity and serorecognition of AMA1 whole proteins and peptides dramatically increased 90 days after AMA1-vaccination in Malian children and adults. a) Bar plots show the 90 day change in seroreactivity among AMA1-vaccinated (n=40, red) and rabies-vaccinated (n=35, blue) Malian children. AMA1 vaccine-allele 3D7 is highlighted in green. AMA1 variants are arranged from greatest to least by the difference in the 90-day change in mean pediatric seroreactivity between AMA1- and rabies-vaccinated Malian children. b) Bar plots show 90 day change in seroreactivity to 263 AMA1 variants among Malian adults who did (red, n=35) and did not (blue, n=19) receive the AMA1 vaccine. Vaccine administration started at the end of the malaria transmission season, and 90 days later corresponded to three months into the dry season of little to no malaria transmission. Vaccine-type AMA1 variant 3D7 is highlighted in green. AMA1 variants are arranged from greatest to least difference in mean 90 day change between AMA1- and control-vaccinated individuals. c) Box and whisker plots show the median and interquartile range of mean seroreactivity to 263 AMA1 variants in Malian adults (n=18), and Malian children (n=40), pre- (blue) and post-vaccination (red) (*p<0.001, Wilcoxon sign-rank test). d) Dot plots represent median and interquartile range of the percent serorecognition of AMA1 whole-protein variants pre- (blue) and 90 days post- (red) AMA1 vaccination in Malian children (n=40) and adults (n=19) (*p<0.001, Wilcoxon sign-rank test). e) Bar plots show the median and interquartile range of seroreactivity to AMA1 peptides in 10 Malian children pre- (blue) and 90 days post- (red) AMA1 vaccination separated by domain (*p<0.01, Wilcoxon sign-rank test). f) Dot plots show the median and interquartile range of percent serorecognition of AMA1 peptides in 10 Malian children pre- (blue) and 90 days post- (red) AMA1 vaccination separated by domain (*p<0.01, Wilcoxon sign-rank test).

Supplementary Fig. S4.

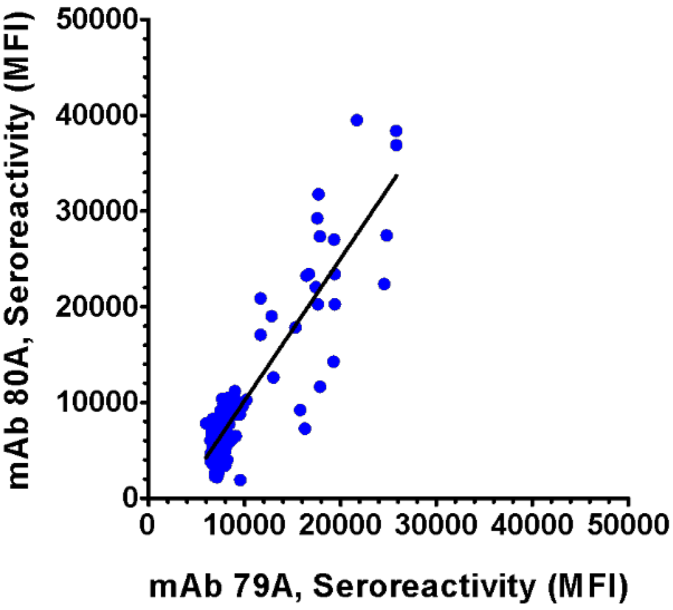

Supplementary Fig. S4. Monoclonal antibodies MRA-479A and MRA-480A bind similarly and specifically to 263 AMA1 variants. Correlation plot shows strong positive correlation between monoclonal antibodies MRA-479A (x-axis) and MRA-480A (y-axis). ($p<0.0001$, Pearson's $r=0.88$, $r2=0.79$)

Supplementary Fig. S5.

A

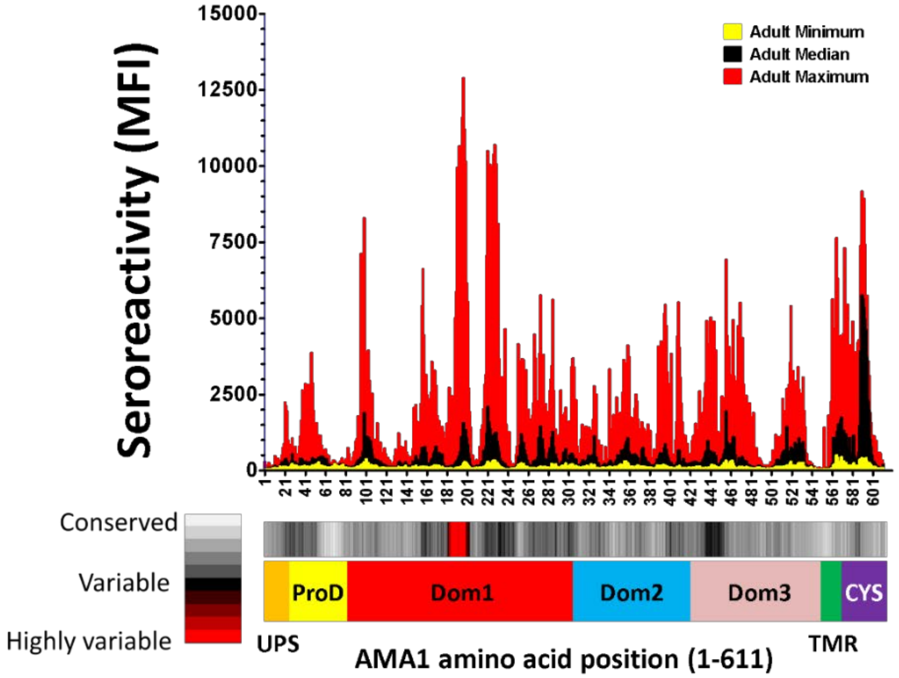

B

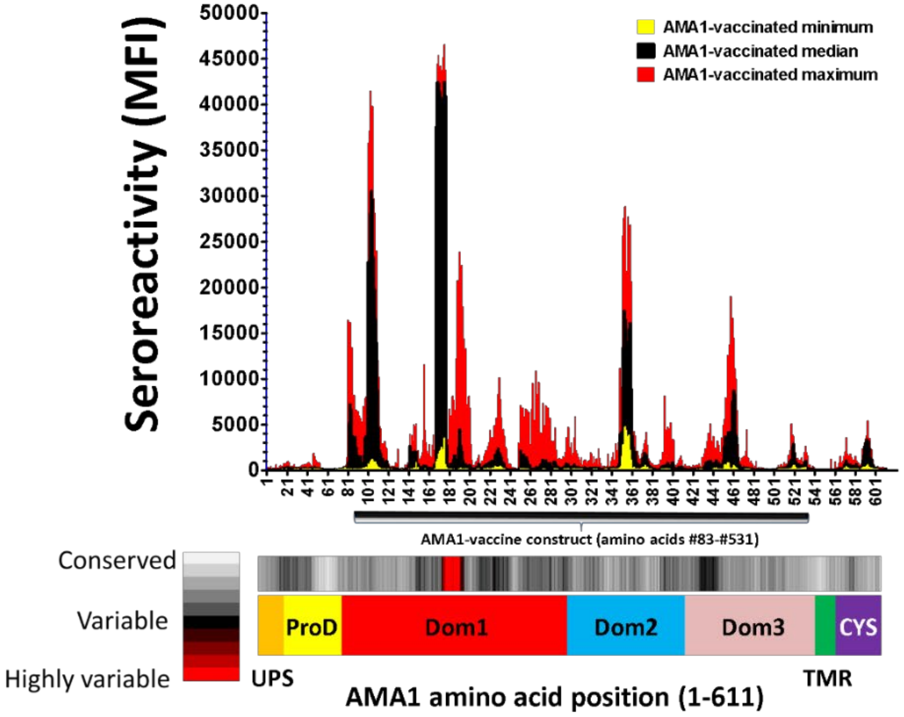

Supplementary Fig. S5. Seroreactivity to AMA1-peptide variants is highly variable among Malian adults. Increases in seroreactivity to AMA1 peptides 90 days after AMA1 vaccination is strain specific in Malian children. a) AMA1 peptide seroprofile bar plots display the average minimum (yellow), median (black), and maximum (red) seroreactivity for each 16-mer peptide at amino acid positions 1-611, for diverse (red) and conserved (gray) regions of AMA1 in Malian adults (n=10). b) Bar plots shows the average minimum (yellow), median (black), and maximum (red) seroreactivity for each 16-mer peptide at amino acid positions 1-611, for diverse (red) and conserved (gray) regions of AMA1 in AMA1-vaccinated Malian children, 90 days after vaccination (n=10).

Data files (separate Excel files)

Supplementary Data file S1: AMA1 protein sequences

Supplementary Data file S2: AMA1 peptide sequences
